# Implementation of online classes during national school closure due to COVID‐19 and mental health symptoms of adolescents: A cross‐sectional survey of 5000 students

**DOI:** 10.1002/pcn5.17

**Published:** 2022-06-22

**Authors:** Ryo Morishima, Haruna Koike, Akiko Kanehara, Kaori Usui, Naohiro Okada, Shuntaro Ando, Kiyoto Kasai

**Affiliations:** ^1^ Department of Neuropsychiatry, Graduate School of Medicine The University of Tokyo Tokyo Japan; ^2^ The Health Care Science Institute Tokyo Japan; ^3^ International Research Center for Neurointelligence (WPI‐IRCN) The University of Tokyo Institutes for Advanced Study (UTIAS) Tokyo Japan

**Keywords:** adolescents, COVID‐19, mental health, national school closure, online classes

## Abstract

**Aim:**

Online classes were implemented in numerous schools during the school closure due to COVID‐19. The present study examined the relationship between online classes during national school closure and mental health symptoms after the reopening of schools.

**Methods:**

We conducted a cross‐sectional survey from October 1 to November 7, 2020 using an anonymous self‐reported questionnaire to evaluate 21 junior and senior high schools in the Saitama prefecture of Japan. Out of the 5538 students who were recruited, 5000 agreed to participate. The relationship between the implementation of online classes and mental health symptoms (emotional symptoms, psychotic experience [PE], and smartphone addiction) was evaluated using mixed‐effect logistic regression models, while controlling for individual and class‐level covariates (e.g., gender, grades).

**Results:**

Implementation of online classes was reported by 78.2% of classroom teachers, and it was associated with lower rates of emotional symptoms (OR = 0.79, 95% CI = 0.63–0.99, *p* = 0.040) and smartphone addiction (OR = 0.79, 95% CI = 0.65–0.96, *p* = 0.020), but not related to PE (OR = 0.91, 95% CI = 0.61–1.36, *p* = 0.637).

**Conclusions:**

Implementing online classes during the national school closure might have had a potential protective effect for adolescents' mental health symptoms (especially emotional symptoms and smartphone addiction) after the reopening of schools during the ongoing COVID‐19 pandemic.

## INTRODUCTION

During the COVID‐19 pandemic, schools closed worldwide to prevent the spread of the infection. Previous studies have indicated that mental health symptoms in adolescents worsened during school closures.^1^, ^2^, ^3^, ^4^, ^5^, ^6^, ^7^ These mental health symptoms have not improved even since schools were reopened. After schools were reopened, the estimated suicide rate among Japanese youth increased 1.49‐fold compared to the pre‐pandemic period, based on the data of suicides in Japan between November 2016 and October 2020 published by the Ministry of Health, Labour, and Welfare.^8^ The well‐being of children and adolescents may not have improved during the period between school closure and reopening.^9^ The national school closure may have continued to affect students' overall activities (due to reduction of learning content and cancellation of school events), lifestyle, peer relationships, and mental health symptoms even after schools reopened. These findings suggest the need to investigate the factors that contributed to the prevention of mental health symptoms in adolescents during the school closure.

Implementing online classes during the school closure may have had a protective effect on mental health, partly through the maintenance of academic achievement and daily routines and the increase in opportunities for social interaction with peers and teachers.^1^, ^4^, ^10^, ^11^, ^12^, ^13^, ^14^ However, existing studies found mixed results regarding the relationship between online classes and mental health symptoms. Some studies conducted more than 9 months after the pandemic indicated worsened mental health during online classes,^15^, ^16^, ^17^ whereas another study conducted within 6 months reported the opposite result.^18^ These differences may be attributed to the survey period. In addition, research was limited to the local‐level school closure, such as geographical region or school, and a cross‐sectional analysis. In the early phase of the COVID‐19 pandemic, almost all of the schools in Japan were closed from late March to May 2020 and reopened in June 2020 in response to requests from the national and local governments.^19^ No study has examined the relationship between online classes during the national school closure and mental health symptoms after schools were reopened.

The present study focused on emotional symptoms, psychotic experience (PE), and smartphone addiction as mental health symptoms. Previous findings suggested that adverse events, such as natural disasters and social deprivation, are associated with increased mental health symptoms among adolescents.^10^, ^20^, ^21^, ^22^ During the ongoing adverse event, COVID‐19, it is important to investigate whether implementing online classes has a potential protective effect on these mental health symptoms. However, mixed results were reported regarding the relationship between online classes during the local school closure and emotional symptoms and depression or anxiety.^16^, ^17^, ^18^ In addition, no study has examined the relationship between online classes and PE or smartphone addiction. We aimed to investigate the relationship between online classes during the national school closure and mental health symptoms after schools reopened. We hypothesized that online classes during the national school closure would be related to lower rates of later mental health symptoms.

## METHODS

This study employed a cross‐sectional survey design using an anonymous questionnaire to evaluate 21 private schools (*N* = 9 junior and *N* = 12 senior high schools) in Saitama prefecture, which is a large prefecture with a population of approximately 7.3 million. The study period was from October 1 to November 7, 2020, and it was approximately 5–6 months after the national school closure due to the first wave of the pandemic in Japan. Our research was conducted through cooperation with the Association for Saitama Private Junior and Senior High Schools and the heads and administrators of Saitama prefecture. Almost all private junior and senior high schools (≥98%) were closed in Japan.^19^ The rate of school closure in Saitama prefecture was higher than in other prefectures,^19^ probably because Saitama prefecture is located in a relatively high COVID‐19 incidence area in Japan.

Before the day of the survey, we informed the students and their parents about the purpose and ethical considerations of the research via a research letter, and that they could opt out of the study if they did not wish to participate. On the day of the survey, teachers distributed the questionnaires to all students and explained that study participation was voluntary and confidential, and that non‐participation carried no disadvantage. Willingness to participate was verified by obtaining written informed consent. This study was approved by the Ethical Committee of the Faculty of Medicine at the University of Tokyo [Approval No. 2019271NI‐(3)] and was performed in accordance with the ethical standards in the 1964 Declaration of Helsinki and its later amendments. Out of the 5538 students who were recruited, 5000 agreed to participate (response rate = 90.3%).

Classroom teachers were asked about the implementation of online classes during the national school closure (*N* = 133/167; response rate = 79.6%). Possible responses were *yes*, *no*, or *unknown*. For the purpose of the present study, we elected *no* as the reference for statistical analysis.

Emotional symptoms were measured using a subscale of the self‐reported Strengths and Difficulties Questionnaire (SDQ).^23^ The emotional symptoms include five items scored as follows: 0 for “not true,” 1 for “somewhat true,” and 2 for “certainly true.” The total score ranges from 0 to 10 and is dichotomized based on the original “abnormal” cutoff thresholds (≥7).^23^ The reliability and validity of the Japanese version of the self‐reported SDQ have been confirmed.^24^ The original “abnormal” cutoff threshold for the emotional symptoms corresponds to approximately the top 10% of the Japanese adolescent population before the pandemic (https://ddclinic.jp/SDQ/standardvalueinjapan.html), while the Japanese version of the self‐reported SDQ has no standardized cutoff threshold. Therefore, to obtain a finding that helps to compare results across countries during the COVID‐19 pandemic, we employed the original “abnormal” cutoff threshold. PE was assessed using one question that referred to the past year (“Please give the answers about yourself over the past year.”): “Have you ever heard voices that other people cannot hear?” (Auditory hallucination). Possible responses were “no,” “yes, probably,” or “yes.” We defined “yes” as the presence of PE. This self‐reported question about auditory hallucination demonstrates good positive and negative predictive validity, not just for clinical interview‐verifiable auditory hallucination but also for other PEs.^25^ Smartphone addiction was measured using the Smartphone Addiction Scale—Short Version (SAS‐SV).^26^, ^27^ SAS‐SV comprises 10 items scored on a six‐point Likert scale, ranging from “strongly disagree” (1) to “strongly agree” (6). The score was calculated by summing all responses, with a possible range of 10–60. The cutoff value of smartphone addiction is ≥31 and ≥33 for male and female participants, respectively.^26^, ^27^

Covariates included students' grades, gender (“male,” “female” or “other”), class size, and remote consultation for students from classroom teacher during the national school closure. Grades and gender were added as basic demographic factors. Class size was added as an environmental factor concerning the degree of resource concentration in the educational setting for individual students during the COVID‐19 pandemic. A previous study in Japan, before the COVID‐19 pandemic, suggests that a larger class size was associated with lower academic performance, lesser support from teachers, and increased mental health symptoms.^28^ As an indicator of the supports provided by teachers during the national school closure, other than online classes, remote consultation for students from the classroom teacher was included as a covariate. The classroom teachers (*N* = 133) were asked about remote consultation for students during this period (“Did you offer consultation to students online or by phone?”). Possible responses were “yes,” “no,” or “unknown.”

Statistical analyses were conducted using R Version 4.1.2. Responses from students and teachers were linked as multilevel data. Descriptive statistics were calculated for all variables. As the implementation of online classes was a class level variable, we used a mixed effect logistic regression model with multiple imputations to examine the relationship between online classes and mental health symptoms. The mixed effect logistic regression model computed the adjusted odds ratios (OR) and 95% confidence intervals (95% CI), with a classroom as a random intercept. Using the mice package in R, the multiple imputations generated 20 imputed data sets. The mixed‐effect models were conducted using the lme4 package. The significance level was set to *α* = 0.05 for all analyses.

## RESULTS

Table 1 shows the descriptive statistics. Implementation of online classes was reported by 78.2% of classroom teachers. Prevalence of emotional symptoms, PE, and smartphone addiction was 18.9%, 5.8%, and 33.1%, respectively.

**Table 1:** Descriptive statistics of study participants (total *N* = 5000)

			Number of missing
Grades, *N* (%)			0
7th	5	(0.1)	
8th	764	(15.3)	
9th	11	(0.2)	
10th	326	(6.5)	
11th	3605	(72.1)	
12th	289	(5.8)	
Gender, *N* (%)			23
Male	2739	(55.0)	
Female	2214	(44.5)	
Other	24	(0.5)	
Online class, *N* (%), (*N* = 133)			0
Yes	104	(78.2)	
No	27	(20.3)	
Unknown	2	(1.5)	
Remote consultation for students, *N* (%), (*N* = 133)			0
Yes	85	(63.9)	
No	48	(36.1)	
Unknown	0	(0.0)	
Class size, mean (SD), (*N* = 133)	33.3	(8.4)	0
Emotional symptoms, *N* (%)	906	(18.9)	203
Psychotic experience, *N* (%)	287	(5.8)	47
Smartphone addiction, *N* (%)	1611	(33.1)	139

The results of the multiple logistic regression analyses are presented in Table 2. The implementation of online classes by the schools was related to lower rates of emotional symptoms (OR = 0.79, 95% CI = 0.63 to 0.99, *p* = 0.040) and smartphone addiction (OR = 0.79, 95% CI = 0.65 to 0.96, *p* = 0.020), but not PE (OR = 0.91, 95% CI = 0.61 to 1.36, *p* = 0.637).

**Table 2:** Results of mixed‐effects logistic regression models for predicting mental health symptoms

	Emotional symptoms				Psychotic experience				Smartphone addiction			
		95% CI				95% CI				95% CI		
	OR	Lower	Upper	*p*‐value	OR	Lower	Upper	*p*‐value	OR	Lower	Upper	*p*‐value
Online classes												
Yes	**0.79**	**0.63**	**0.99**	**0.040**	0.91	0.61	1.36	0.637	**0.79**	**0.65**	**0.96**	**0.020**
No	1				1				1			
Unknown	0.55	0.26	1.16	0.115	0.81	0.23	2.88	0.743	1.05	0.54	2.02	0.885
*Covariates*												
Grades	**1.10**	**1.02**	**1.18**	**0.016**	**0.88**	**0.79**	**0.98**	**0.016**	**1.16**	**1.09**	**1.24**	**<0.001**
Gender												
Male	1				1				1			
Female	**2.52**	**2.17**	**2.93**	**<0.001**	**1.34**	**1.05**	**1.71**	**0.020**	**0.77**	**0.68**	**0.87**	**<0.001**
Other	**10.73**	**4.59**	**25.11**	**<0.001**	**6.03**	**2.29**	**15.87**	**<0.001**	0.76	0.30	1.92	0.556
Remote consultation for students												
Yes	**1.31**	**1.07**	**1.61**	**0.008**	**1.73**	**1.25**	**2.40**	**0.001**	1.10	0.93	1.31	0.265
No	1				1				1			
Class size	**0.98**	**0.97**	**0.99**	**0.003**	**0.96**	**0.95**	**0.98**	**<0.001**	0.99	0.98	1.00	0.245

*Not*e: Bold represents statistical significance.

Abbreviations: OR, odds ratio; 95% CI, 95% confidence intervals.

In addition, higher grades were associated with higher rates of emotional symptoms and smartphone addiction, but related to lower rates of PE (Table 2).

## DISCUSSION

To the best of our knowledge, this is the first study to investigate the relationship between implementation of online classes during the national school closure and the mental health symptoms after schools reopened. This relationship was observed in emotional symptoms and smartphone addiction, but not in PE.

Online classes were found to be associated with lower rates of some mental health symptoms. Our findings were consistent with those in a study conducted within 6 months after the pandemic.^18^ However, they were not consistent with studies conducted after more than 9 months.^15^, ^16^, ^17^

Additionally, online classes may have had a protective effect on mental health symptoms for school closure at the national level, rather than that at the local level. While there are previous studies indicating adverse effects of online classes conducted during school closure at the local level,^15^, ^16^, ^17^ the present study focused on the effect during school closure at the national level. The difference between the findings could be explained by the conservation of resources (COR) theory. The COR theory posits that the loss of resources is a primary cause of stress, and individuals may feel more stressed when they lose more valuable resources.^29^, ^30^ During the local school closure, students who participated in online classes may have more likely felt that they lost their resources (e.g., learning environment and opportunities for social interaction), compared to those in face‐to‐face classes. However, during the national school closure, students participating in online classes may have felt that they were gaining resources rather than losing them, thus resulting in less stress.

Furthermore, the relationship between online classes and long‐term mental health problems may be partially explained by the disruption of academic performance, daily routines, and social interactions during the pandemic. The national school closure (March–May 2020) coincided with the start time of the academic year (April) in Japan. Previous studies on Japanese adolescents before the COVID‐19 pandemic suggested that poor academic performance, disrupted daily routines, and fewer social interactions were associated with increased depressive and anxiety symptoms.^31^, ^32^, ^33^ Future studies are needed to examine whether these relationships were strengthened by the pandemic.

In addition, older adolescents were more likely to have emotional symptoms and smartphone addiction, but less likely to have PE, compared to younger adolescents. These results were in line with the findings on the pre‐pandemic^34^, ^35^, ^36^, ^37^ and post‐pandemic periods.^4^, ^5^, ^12^, ^38^ However, the increasing trend of mental health symptoms between the pre‐ and post‐pandemic periods was more substantial, particularly in young age groups than in other age groups.^38^ Thus, further provision of developmentally appropriate support for youth may be needed during the COVID‐19 period.

This study has several implications for researchers, clinicians, and educators. Future research needs to examine whether the effect of online classes on mental health symptoms depends on the phase of pandemic or level of school closure. It is also necessary to investigate whether the lack of online classes is related to later mental health symptoms mediated by academic performance, daily routines, and social interaction. We believe that our study provided valuable findings for the school community through the cooperation with schoolteachers of the Association for Saitama Private Junior and Senior High School. However, given the discussion with the teachers, we did not include some important items for adolescent mental health (e.g., maltreatment, substance abuse, self‐harm, suicidality) in the questionnaire. Further study is required to investigate mental health symptoms of adolescents, including these items, during the national school closure. Policymakers and school administrators could consider implementing online classes during national school closure owing to its potential protective effect on mental health. Students for whom online classes were not implemented during the national school closure should be screened for mental health symptoms, especially emotional symptoms and smartphone addiction, after schools reopen.

The present study is limited in that it is a cross‐sectional study design. A longitudinal study is needed to evaluate a causal relationship between online classes during national school closure and later mental health symptoms. The second limitation is that the PE item in the present study could not adequately distinguish the pre‐pandemic experience, and it was used to measure auditory hallucination only. A future study is needed to examine the relationship between the implementation of online classes and PE, which should be limited to post‐pandemic experiences and include other common aspects of PE, such as persecutory thoughts, visual hallucinations, thought broadcasting, and special messages.

In conclusion, this is the first study to indicate that implementation of online classes during the national school closure due to the COVID‐19 pandemic was related with lower rates of mental health symptoms after schools reopened. The relationship was observed in emotional symptoms and smartphone addiction, but not in PE. The findings suggested that implementation of online classes during the national school closure is one of the measures to prevent some mental health symptoms. Further research is required to examine whether the protective effect depends on the pandemic phase or level of school closure, and to identify the mediators in this relationship.

## AUTHOR CONTRIBUTIONS

Ryo Morishima, Haruna Koike, Akiko Kanehara, Kaori Usui, Naohiro Okada, Shuntaro Ando, and Kiyoto Kasai conceptualized and designed the study. Ryo Morishima, Haruna Koike, Akiko Kanehara, Kaori Usui, and Kiyoto Kasai collected the data. Ryo Morishima and Kiyoto Kasai acquired funding. Ryo Morishima conducted the statistical analyses. Ryo Morishima wrote the first draft of the manuscript. All authors contributed to and have approved the final manuscript.

## CONFLICT OF INTEREST

The authors declare no conflict of interest.

## ETHICS APPROVAL STATEMENT

This study was approved by the Ethical Committee of the Faculty of Medicine at the University of Tokyo [Approval No. 2019271NI‐(3)] and was performed in accordance with the ethical standards in the 1964 Declaration of Helsinki and its later amendments.

## PATIENT CONSENT STATEMENT

Participation was voluntary, confidential, and carried no disadvantage for non‐participation. Willingness to participate was verified by obtaining written informed consent.

## Data Availability

The availability of the data in this study is not open access due to the provisions of the ethics committee and the extent of the participants' consent. If readers wish to apply for the use of data, they must contact the corresponding author and consult the Ethics Committee, Faculty of Medicine, The University of Tokyo.
